# Validation, optimisation, and application data in support of the development of a targeted selected ion monitoring assay for degraded cardiac troponin T

**DOI:** 10.1016/j.dib.2016.02.051

**Published:** 2016-03-03

**Authors:** Alexander S. Streng, Douwe de Boer, Freek G. Bouwman, Edwin C.M. Mariman, Arjen Scholten, Marja P. van Dieijen-Visser, Will K.W.H. Wodzig

**Affiliations:** a Central Diagnostic Laboratory, Maastricht University Medical Centre, Maastricht, the Netherlands; b Department of Human Biology, Maastricht University, Maastricht, the Netherlands; c Biomolecular Mass Spectrometry and Proteomics, Bijvoet Centre for Biomolecular Research and Utrecht Institute for Pharmaceutical Sciences, Utrecht University, Utrecht, the Netherlands; d Netherlands Proteomics Centre, Utrecht University, Utrecht, the Netherlands; e Current working address: Janssen, Pharmaceutical Companies of Johnson&Johnson, Infectious Diseases and Vaccines, Leiden, the Netherlands

## Abstract

Cardiac troponin T (cTnT) fragmentation in human serum was investigated using a newly developed targeted selected ion monitoring assay, as described in the accompanying article: “Development of a targeted selected ion monitoring assay for the elucidation of protease induced structural changes in cardiac troponin T” [1]. This article presents data describing aspects of the validation and optimisation of this assay. The data consists of several figures, an excel file containing the results of a sequence identity search, and a description of the raw mass spectrometry (MS) data files, deposited in the ProteomeXchange repository with id PRIDE: PXD003187.

## Specifications table

**Table:** 

Subject area	*Clinical chemistry.*
More specific subject area	*Cardiovascular biomarkers.*
Type of data	*Figures, graphs, table, MS data.*
How data was acquired	*SDS-PAGE with Coomassie staining, MS data acquired with the Q Exactive (Thermo Scientific), and an online database search.*
Data format	*Raw data (.raw, .msf, .xlsx) and analysed data (figures).*
Experimental factors	*cTnT fragments were created by spiking intact cTnT in human serum and incubation at 37 °C for varying amounts of time.*
Experimental features	*Intact and fragmented cTnT was purified by immunoprecipitation, separated by SDS-PAGE, manually excised and digested with trypsin followed by relative quantification using targeted-SIM.*
Data source location	*Maastricht, the Netherlands.*
Data accessibility	*All figures are provided in this article. MS.raw- and.msf-files are deposited in the ProteomeXchange repository with id PRIDE: PXD003187.*

## Value of the data

• The mass spectrometry data identifies multiple protein bands present in human serum as cardiac troponin T.
• Our approach to relate the intensity of precursor ions of interest to a reference ion within the same sample is a useful tool for gel-based targeted proteomics.
• Our approach to identify changes within a peptide can be used to accommodate other proteins and modifications.
• Our data about tryptic cTnT precursor ions is invaluable to researchers studying the same protein.

## 1 Data

**Fig. 1:**
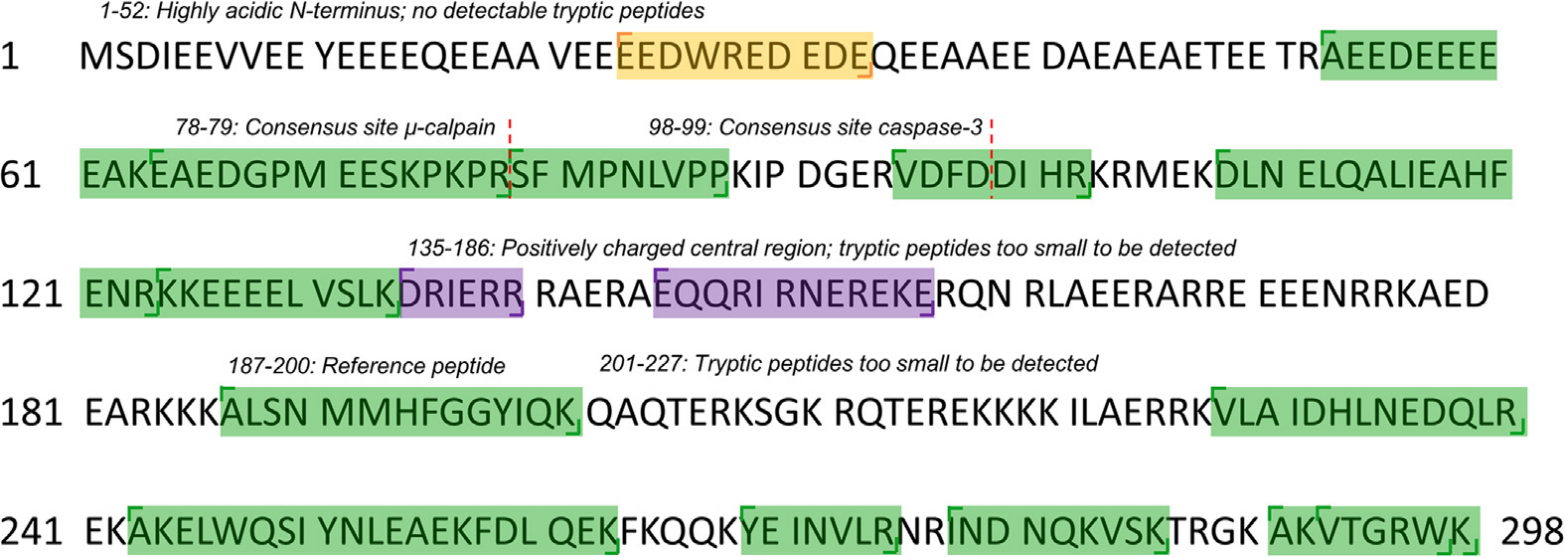
Annotated sequence of the canonical protein species of cardiac troponin T (cTnT). Green highlights indicate peptides of interest that were synthesised and pooled in the synthetic peptide standard. The orange highlighted area is spliced in the human adult cTnT protein species (isoform 6). The purple highlighted areas indicate the target epitopes of the clinical cTnT assay by Roche Diagnostics. (For interpretation of the references to color in this figure legend, the reader is referred to the web version of this article.)

**Fig. 2:**
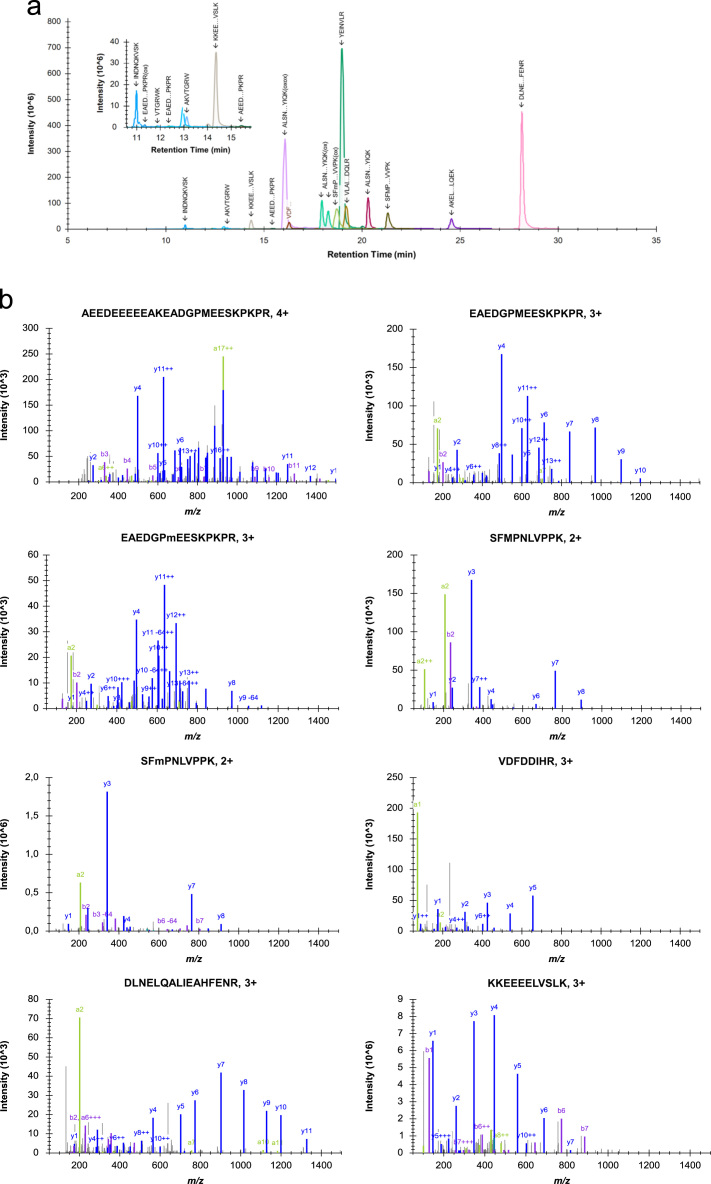

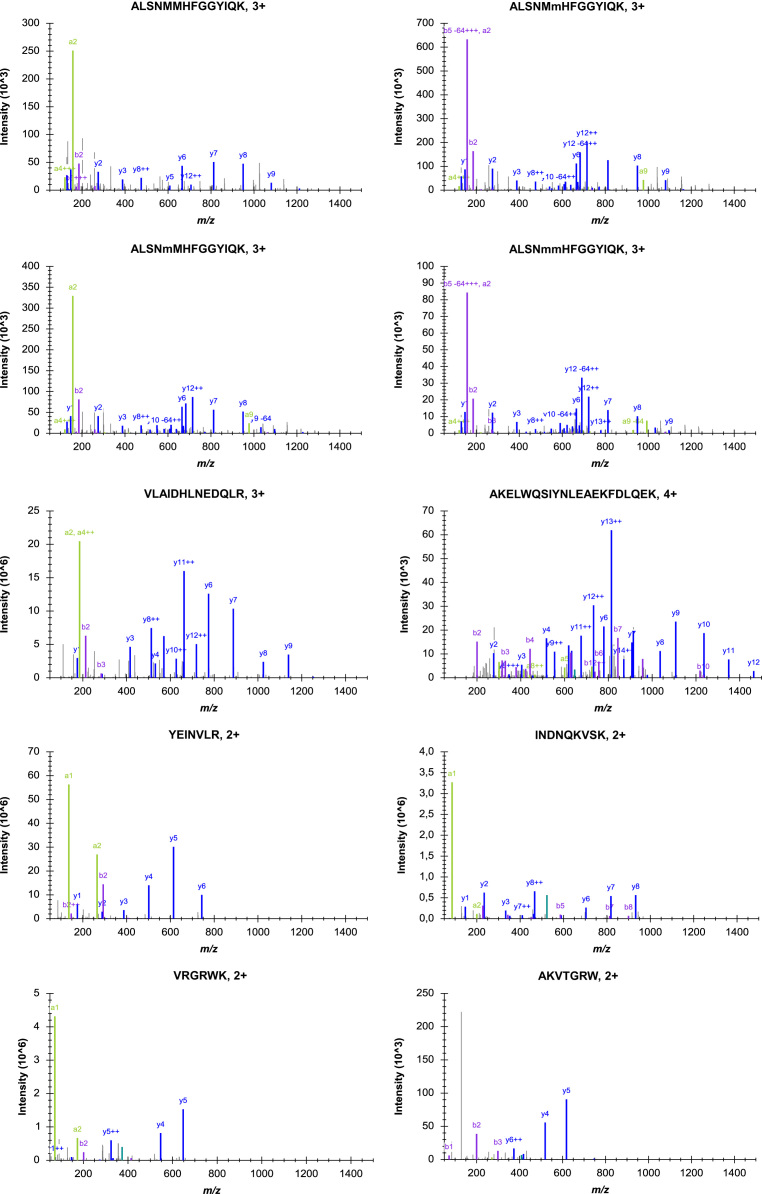
a. Selected ion chromatogram of all targeted precursor ions as present in the synthetic standard. Inset shows the area between 10.8 and 15.4 min containing several poorly ionisable precursor ions. b**.** Annotated MS/MS spectra identifying each of the targeted precursor ions in the synthetic standard.

**Fig. 3:**
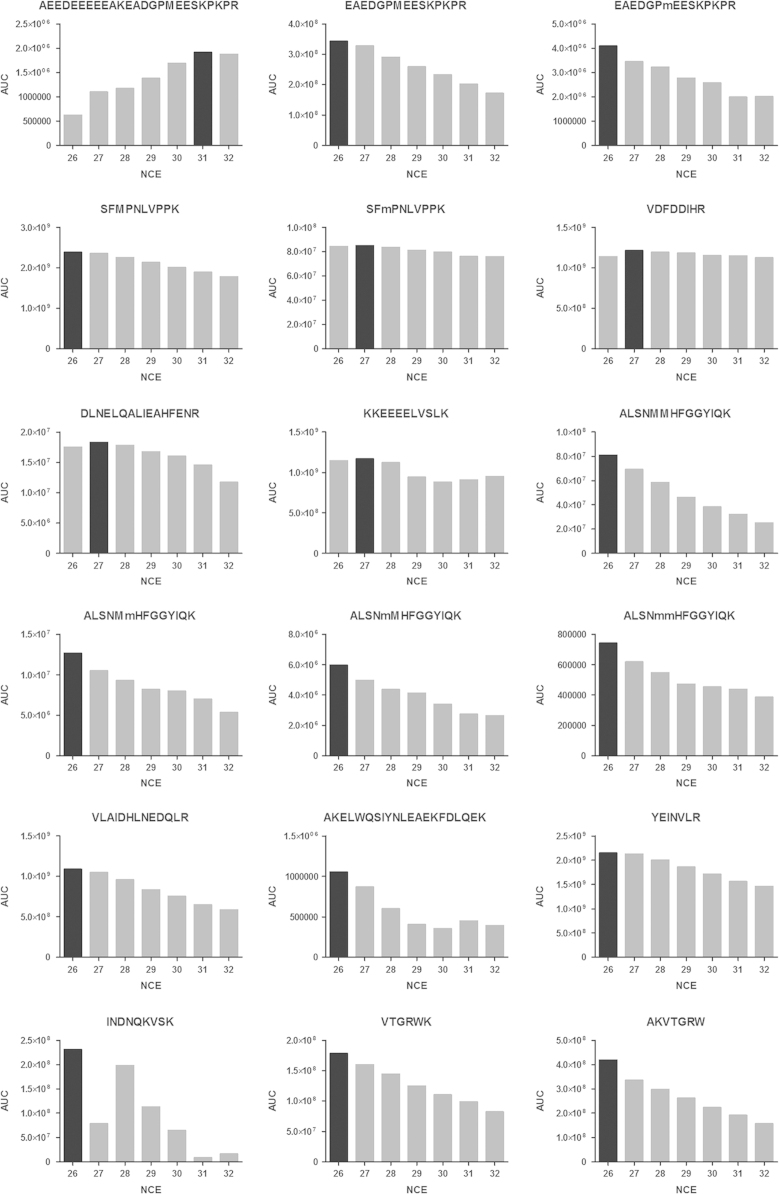
Normalised collision energy (NCE) optimization of the selected precursor ions. MS2 spectra were recorded at normalised collision energies varying between 26 and 32. The collision energy setting resulting in the highest signal (black) was chosen for future experiments. AUC denotes area under the curve.

**Fig. 4:**
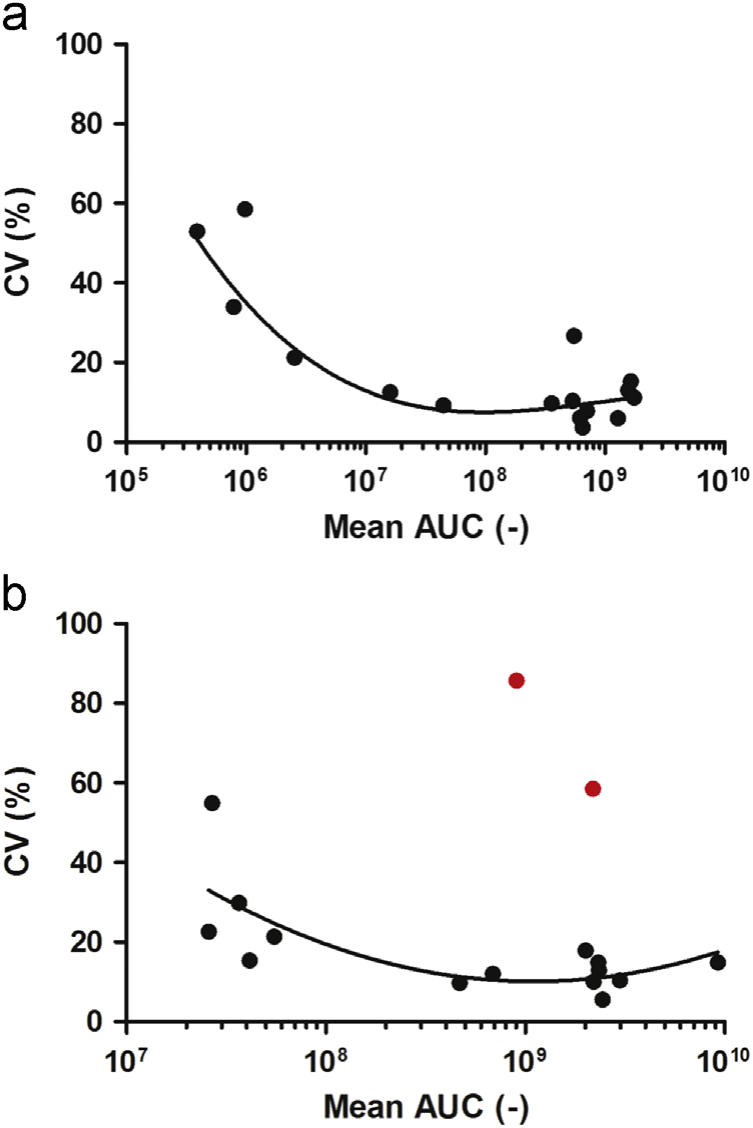
a**.** Precision plot depicting the instrument CV of the Q Exactive mass spectrometer versus the mean AUC of 16 selected precursor ions of cTnT. Six technical replicates were injected into the mass spectrometer. b**.** Precision plot depicting the total CV of the entire experimental workflow versus the mean AUC of 16 selected precursor ions of cTnT. Six technical replicates were individually processed and injected into the mass spectrometer. Red data points indicate precursor ions with abnormally high CV. (For interpretation of the references to color in this figure legend, the reader is referred to the web version of this article.)

**Fig. 5:**
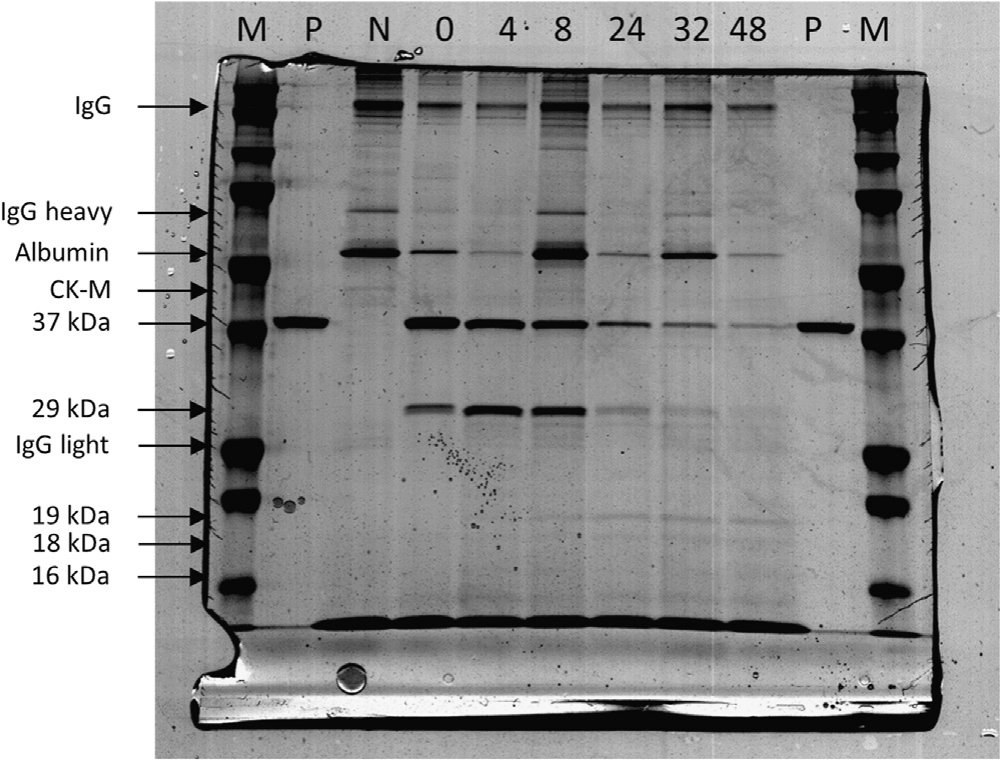
Coomassie-stained gel image of incubated serum samples spiked with cTnT after immunoprecipitation. This gel was used to excise protein bands for t-SIM analysis. cTnT bands at 37, 29, 19, 18, and 16 kDa are marked. Several common coeluting proteins are also marked. IgG, immunoglobulin G; CK-M, creatine kinase M-type; M, molecular weight marker; P, positive control; N, negative control.

The data in this article supports the validation and optimisation of a targeted selected ion monitoring (t-SIM) assay used to analyse an observed fragmentation pattern of the protein cardiac troponin T (cTnT) [1]. Fig. 1 depicts the amino acid sequence of cTnT with the targeted peptides of interest highlighted in green, Fig. 2 shows the selected ion chromatogram and the MS/MS identification of these peptides, Fig. 3 shows the result of a collision energy optimisation experiment performed on each single peptide, Fig. 4 shows precision plots of the validated assay, and Fig. 5 depicts a Coomassie-stained image of a gel prior to the application of the finalised method. All related mass spectrometry data is also publicly available via the ProteomeXchange repository (PRIDE: PXD003187).

## 2 Experimental design, materials and methods

### 2.1 Peptide specificity

The specificity of each targeted peptide for cTnT was verified by performing a sequence identity search of all included peptides using the peptide match tool of the online Protein Information Resource (PIR, http://research.bioinformatics.udel.edu/peptidematch/index.jsp) [2], [3]. The database that was searched was the complete, unrestricted, UniprotKB database from September 2014 (84,539,639 sequences). Leucine and isoleucine residues were considered equivalent. The complete search results are publicly available in the online data supplement (Sequence identity search.xlsx).

### 2.2 Retention time approximation

The targeted cTnT peptides of interest (Fig. 1) were synthesised by Pepscan (Lelystad, the Netherlands). The synthesised peptides were combined in equimolar amounts to create a synthetic peptide standard.

This synthetic peptide standard was analysed with a data dependent (Top 10) method on a Q Exactive hybrid quadrupole-Orbitrap mass spectrometer, connected to a UHPLC Proxeon Easy-nLC 1000 by Thermo Scientific (Waltham, MA, USA). Peptides were first trapped on an Acclaim PepMap 100, 100 µm x 2 cm, C18, 5 µm, 100 A trap column in 0.1% TFA, 2% ACN and 98% water. Peptides were subsequently separated on an Acclaim PepMap RSLC, 75 µm x 15 cm, C18, 2 µm, 100 A analytical column by a 30 min gradient of 4–55% buffer B, followed by 55–90% B in 1 min and 90% B for 4 min at a flow rate of 300 nL/min.

Full scan MS spectra were acquired in the Orbitrap in the m/z range 250–1500 at a resolution of 70,000 full width at half maximum (FWHM) at 200 m/z, automatic gain control (AGC) of 1,000,000, and a maximum injection time of 250 ms. The 10 most intense precursor ions were selected for higher-energy collisional dissociation (HCD) with an isolation window of 1.2 Th and a normalised collision energy (NCE) of 33%. Product ions were detected in the m/z range 250–1500 at a resolution of 17,500 FWHM, AGC target of 100,000, maximum injection time of 200 ms, and a dynamic exclusion window of 60 s.

Fig. 2a depicts the selected ion chromatogram of the synthetic peptide standard and shows the retention times of all targeted peptides. MS/MS identifications of the targeted peptides are provided in Fig. 2b. Obtained retention times from this data can be used to schedule targeted measurements on the depicted cTnT precursor ions when using a similar chromatography setup and gradient. When doing multiple experiments, shifts in retention time may be observed. It is therefore advisable to acquire the current retention time of each target by measure the peptide standard prior to analysing biological samples.

### 2.3 Normalised collision energy optimisation

Normalised collision energies (NCE) were optimised using the synthetic peptide standard and a targeted MS2 (PRM) method. The same chromatographic settings were used as described in Section 2.2. Previously observed retention times and m/z values were used to target and isolate precursor ions of interest (isolation window 1.2 Th) for HCD fragmentation at a resolution of 35,000, AGC of 100,000, and a maximum injection time of 200 ms. NCE was varied between 26 and 32.

Integration of the product ion chromatograms was performed on the 6 most abundant fragment ions using Skyline version 2.6 [4]. The total area under the curve (AUC) for each precursor ion then equals the sum of the AUCs of the 6 most abundant fragment ions. The data in Fig. 3 depicts the total AUC for each precursor ion at the different NCE settings. The NCE setting with the highest total AUC for each precursor ion was used in future MS/MS measurements.

### 2.4 Analysis of technical replicates

Coefficient of variation (CV) was calculated for the instrument by injecting the synthetic peptide standard 6 times while using a t-SIM method. Additionally, the CV was calculated for 6 cTnT in-gel digests prepared according to the workflow in [1]. The same chromatographic settings were used as described in Section 2.2. SIM was performed on previously observed m/z values and retention times at a resolution of 70,000 FWHM at m/z 200, AGC of 100,000, maximum injection time of 250 ms and a detection window of 2.0 Th. The total AUC for each precursor ion equals the sum of the AUC of its M, M+1 and M+2 isotopologues, as determined using Skyline version 2.6. From the data, precision plots were created for the Q Exactive instrument (Fig. 4a) and for the experimental workflow described in [1] (Fig. 4b.).

### 2.5 Sample preparation

The validated t-SIM assay is applied on serum samples spiked with purified human cTnT and incubated at 37 °C. Prior to t-SIM analysis, cTnT is captured from serum using an immunoprecipitation technique employing the M11.7 catcher antibody by Roche Diagnostics (Basel, Switzerland) based on a protocol by Michielsen et al. [5]. This is followed by gel electrophoresis and Coomassie staining. Fig. 5 depicts the Coomassie stained gel with several protein bands marked. In-gel digestion of the bands marked 37, 29, 19, 18, and 16 kDa can be performed to obtain samples suitable for relative quantification using this t-SIM assay.

### 2.6 Direct link to deposited data

Mass spectrometry data (.raw-files and.msf-files) from the validation of this assay and data from the application of the assay on samples obtained in Section 2.5 and [1] are deposited to the ProteomeXchange Consortium (http://proteomecentral.proteomexchange.org) via the PRIDE partner repository with the dataset identifier PRIDE: PXD003187 [6], [7].
