# Supplementary material for: Validation, optimisation, and application data in support of the development of a targeted selected ion monitoring assay for degraded cardiac troponin T
Source: Data Brief. 2016 Mar 3;7:397–405. doi: 10.1016/j.dib.2016.02.051 (PMC4782000; doi:10.1016/j.dib.2016.02.051)
Supplement: Supplementary file 1 — Supplementary material [file mmc1.docx]

**Conflicts of interest**

This study was funded in part by a grant from Stichting de Weijerhorst to M.P. van Dieijen-Visser. It was supported by the PRIME-XS project, grant agreement number 262067, which is funded by the European Union Seventh Framework Program. The Netherlands Proteomic Centre, embedded in the Netherlands Genomics Initiative, is acknowledged for funding. Roche Diagnostics kindly provided the monoclonal M7 and M11.7 antibodies for use in immunoprecipitation and Western blot experiments. A. Scholten performed this work prior to his employment with Janssen, Pharmaceutical Companies of Johnson&Johnson and it has no relation to his current work at Janssen.
